# Contralateral supracerebellar transtentorial approach for a thalamic cavernous malformation resection: operative video

**DOI:** 10.3171/2019.7.FocusVid.19187

**Published:** 2019-07-01

**Authors:** Sirin Gandhi, Tsinsue Chen, Justin R. Mascitelli, Claudio Cavallo, Mohamed A. Labib, Michael J. Lang, Michael T. Lawton

**Affiliations:** Department of Neurosurgery, Barrow Neurological Institute, St. Joseph’s Hospital and Medical Center, Phoenix, Arizona

**Keywords:** cavernous malformation resection, contralateral approach, supracerebellar transtentorial approach, thalamic cavernous malformation, video

## Abstract

This video illustrates a contralateral supracerebellar transtentorial (cSCTT) approach for resection of a ruptured thalamic cavernous malformation in a 56-year-old woman with progressive right-sided homonymous hemianopsia. The patient was placed in the sitting position, and a torcular craniotomy was performed for the cSCTT approach. The lesion was resected completely. Postoperatively, the patient had intact motor strength and baseline visual field deficits with moderate right-sided paresthesias. The cSCTT approach maximizes the lateral surgical reach without the cortical transgression seen with alternative transcortical routes.^1^ Contralaterality is a defining feature, with entry of the neurosurgeon’s instruments from the craniotomy edge of the craniotomy, contralateral to the lesion, allowing access to the lateral aspect of the lesion. The sitting position facilitates gravity-assisted cerebellar retraction and enhances the superior reach of this approach (Used with permission from Barrow Neurological Institute, Phoenix, Arizona).

The video can be found here: https://youtu.be/lqB9mu_T8NQ.

## Transcript

This video will demonstrate the contralateral supracerebellar transtentorial approach for a thalamic cavernous malformation resection.

### 0:29 Clinical presentation

The patient is a 56-year-old woman who presented with progressive vision loss. On exam, she had a right homonymous hemianopsia. She was otherwise neurologically intact.

### 0:41 Preoperative imaging

Here are her MRI pictures demonstrating the lesion in the lateral aspect of the thalamus. You can see surrounding edema on these T2-weighted images, indicating recent hemorrhage. On the coronal images, you can see how it is inaccessible from traditional transventricular or interhemispheric approaches as well as quite deep for transsylvian transinsular approaches.

### 1:05 Surgical strategy and patient positioning

A supracerebellar transtentorial approach was selected. This is done in the sitting position in order to allow gravity to retract the cerebellum and gain additional superior and lateral trajectories. Here is the intraoperative view, showing the incision in the midline as well as the torcular craniotomy and dural opening. In addition to the dural opening, a cut is made in the tentorium to access the supraincisural space. Here is the intraoperative video showing the cerebellar hemispheres.

### 1:40 Opening of cisterna magna

The first move is to go down to the cisterna magna to drain CSF, and this relaxes the cerebellum and allows this cerebellar sagging. There are typically one or two bridging veins from the superior surface of the cerebellum to the tentorium, which are easily cut.

### 1:58 Neuronavigation

Here the Stealth navigation shows the trajectory which takes us all the way to the Galenic region. Now as the dissection proceeds, the arachnoid around the tectum is opened widely. The dissection is now proceeding lateral to the collicular plate to the left side. You can see the medial occipital lobe and branches of the posterior cerebral artery here, falling into the quadrigeminal cistern. The dissection here with microscissors is being performed to mobilize the occipital lobe as far laterally as possible, but you can see that there is a limit due to the tentorial incisura.

### 2:40 Incision of contralateral tentorium

Therefore, an incision is made in the tentorium to relax this structure and enable additional lateral retraction. So, here is that notch created in the tentorium, and you can see now with the probe how we can then exceed or extend beyond the incisura. We have a great view of the basal vein of Rosenthal as well as the PCA as they wrap around from the quadrigeminal cistern into the ambient cistern. Here is the entry point into the undersurface of the thalamus. Just below this cortical incision, this old hemorrhage cavity is entered.

### 3:16 Evacuation of hemorrhagic clot

First, the clot is evacuated, and as clot is evacuated, we can now begin to see the caverns of the malformation. We can then begin to work them free of their adhesions to the adjacent thalamus.

### 3:32 Piecemeal resection of cavernous malformation

I get inside of the capsule of the malformation first and take these little pockets of clot and fluid in order to create working space internally. Here is a piece of that internal portion of the lesion. You will notice that I am working from the patient’s right side and crossing over to the left, and this is what is creating that cross-court trajectory. Now you can see the planes of the dissection cavity being opened and pieces of the malformation removed bit by bit. Here is a large piece coming out, after having freed this circumferentially on all sides with a series of round knives and some gentle traction. Also note that I am using a lighted suction, which provides light within the cavity, and now we are seeing normal gliotic planes on all sides. There is no evidence of any additional cavernous malformation there, and you can see from Stealth how we have reached the deepest extent of the lesion. Here is an overview showing that crossing trajectory that additional room created by the transtentorial cut and the upward trajectory into the thalamus from below.

### 4:44 Postoperative outcome

The patient had good strength postoperatively but did have some new numbness in the right arm and leg that was transient. She was discharged to rehab on postoperative day 3, and on follow-up examination her strength and her sensory function had returned to normal.

### 5:00 Postoperative imaging

Here are her postoperative images showing a complete resection of the lesion. The edema is seen around the resection cavity, but you can see no evidence of any retained malformation.

### 5:14 Conclusion

In conclusion, this contralateral supracerebellar transtentorial approach maximizes the lateral reach of the traditional supracerebellar infratentorial approach. The sitting position facilitates gravity retraction of the cerebellum and extends the superior reach of the supracerebellar infratentorial approach. This approach is far less invasive than transcortical routes from the cerebral convexity, like the superior parietal lobule approach. The proximity to the internal capsule on the anterior surface of the lesion must be appreciated and, in this case, accounted for her transient postoperative sensory changes.
